# Quality of life and health care consumption in primary care patients with elevated serum calcium concentrations in - a prospective, case control, study

**DOI:** 10.1186/1471-2296-15-84

**Published:** 2014-05-05

**Authors:** Sofia Dalemo, Robert Eggertsen, Per Hjerpe, Svante Jansson, Kristina Bengtsson Boström

**Affiliations:** 1 Institute of Medicine/Department of Public Health and Community/Primary Health Care, Sahlgrenska Academy, University of Gothenburg, Gothenburg, Sweden; 2 R&D Centre, Skaraborg Primary Care, Skövde, Sweden; 3 Mölnlycke Primary Health Care and Research Centre, Gothenburg, Sweden; 4 Institute of Clinical Sciences/Department of Surgery, Sahlgrenska Academy, University of Gothenburg, Gothenburg, Sweden

**Keywords:** Hypercalcaemia, Primary care, Mortality, Quality of Life, SF-36, Gender, Sick leave

## Abstract

**Background:**

Patients with elevated calcium concentrations have an increased morbidity due to various underlying illnesses. However, there is a lack of studies of quality of life and health care consumption in patients with hypercalcaemia per se. The study aims to investigate quality of life and health care consumption, as measured by, sick leave, drug prescriptions and the number of visits and admissions to health care centres and hospitals, in primary care patients with elevated calcium concentrations.

**Methods:**

A prospective, case control, study in primary care centre, in Sweden. Patients with elevated, (n = 127, 28 men), and normal calcium concentrations, (n = 254, 56 men), mean age 61.4 year, were recruited in the study and followed during 10 years. Eighty-six percent of those alive at the time of follow up participated in a follow up visit. The study participants completed a quality of life survey, SF-36, which also were compared with the Swedish SF-36 national normative database.

**Results:**

Patients with elevated calcium concentrations had significantly lower quality of life both compared with the control group (patients with normal calcium concentrations) and compared with age and gender-matched reference material from the Swedish SF-36 national normative database. The group with elevated calcium concentrations had significantly more hospitalisations (p = 0.017), subsequently cancer diagnoses (p < 0.003), sick leave (p = 0.007) and medication (p = 0.002) compared with patients with normal calcium concentrations. Men with elevated calcium concentrations had more contacts with the psychosocial team (p = 0.02) at the health care centre.

**Conclusions:**

Elevated calcium concentrations are associated with significantly reduced quality of life and increased health care consumption and should therefore be an important warning flag that should alert the physician to further investigate and care for the patient. This is the first study in this field and the results need to be confirmed in further studies.

## Background

Calcium analysis, in plasma or serum, has been used in primary care for a long time in the screening for primary hyperparathyroidism, (pHPT), and malignant diseases. Screening is useful since symptoms are insidious and patients with increased calcium concentrations do not always realise that their symptoms can be a sign of disease, such as pHPT [1]. When point of care analyses of calcium were introduced in primary health care centres, (PHCCs) some decades ago the number of analyses increased [2]. From previous studies it is known that calcium concentrations in primary care patients are often just moderately elevated [2]. We have previously shown that at least 88% of patients with elevated calcium concentrations to have an underlying disorder [2].

The most common cause of elevated calcium concentration in primary care is pHPT. The increased calcium screening has resulted in that pHPT patients are diagnosed earlier, when they have milder disease The clinical picture of pHPT, has therefore changed during recent years from critically ill patients to patients with subtle symptoms such as depressed mood, confusion, fatigue, sleep disorders, neuromuscular symptoms or less obvious symptoms mainly diagnosed in primary care [3-5]. However, the severity of psychiatric symptoms is not linearly related to the degree of hypercalcaemia [6]. pHPT patients experience a reduced quality of life, QoL, [7,8]. A Swedish screening study showed that women with a formerly unknown mild form of pHPT visited physicians more often and had increased sick leave in the years prior to the diagnosis [9,10]. More recently, an increased total mortality from cardiovascular disease [11] and cancer [2] has been found in men with hypercalcaemia*.* However, there is a lack of studies of QoL in patients with hypercalcaemia per se, and it is unknown how this is reflected in health care consumption for this group of patients.

The primary objective of this study was to investigate QoL in adult patients with elevated calcium concentrations, in order to provide guidance to further management in primary care. The secondary objective was to study whether elevated calcium concentrations affect health care consumption, as measured by sick leave, drug prescriptions and the number of visits and admissions to health care centres and hospitals.

## Methods

### Subjects

**Figure 1:**
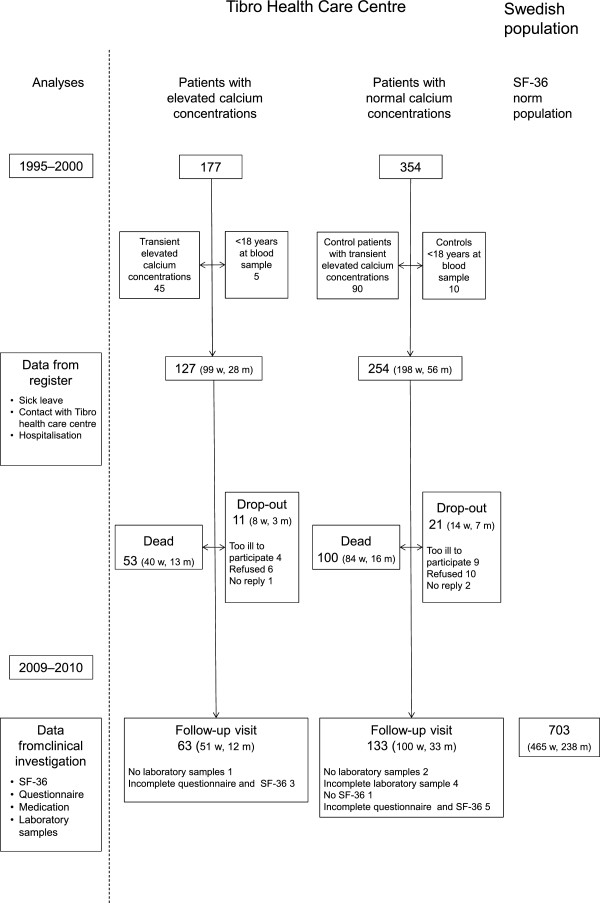
**Included patients and variables.** Flow chart of drop-outs and included variables among patients with elevated and normal calcium concentrations at Tibro Health Care Centre, Sweden, first investigation1995–2000, and re-examination 2009–2010.

Tibro is a rural community in Sweden with 11,000 inhabitants and one PHCC. Medical records of all patients with elevated calcium concentrations between 1995–2000 have been studied previously [12], Figure 1. At baseline, 1995–2000, an elevated calcium concentration was defined as ≥2.56 mmol/l in at least one test. Calcium was corrected for albumin in 75% of patients who had taken it in conjunction with the calcium sampling. Two age and sex-matched controls with calcium < 2.45 mmol/l were selected for each patient from the PHCCs electronic data. Age was matched within 2 months, but for the oldest (n = 2), the match was within three years.

Individuals with borderline calcium concentrations, between 2.45- 2.55 mmol/L, were excluded to avoid values difficult to interpret. For 45 patients there was only an initial single high calcium concentration. The majority of these samples were analysed between January 1997 to April 1998, when quality controls revealed that the analyses deviated above defined quality levels [2]. At follow up these patients had no underlying diagnoses and were therefore excluded from the analyses, Figure 1.

For all patients, data regarding visits to general practitioner and psychosocial team between 1st January 1998 and 8th December 2010 and certified sick leave between 4th November 2003 and 27th December 2010 was collected from their health care medical records (ProfDoc Journal III, ProfDoc AB). Diagnoses and durations of all inpatient care between 1st January 1967 and 31st December 2010 were derived from The Swedish National Board of Health and Welfare. The different time intervals were due to variation of registration in the registries.

All study subjects alive at the time of follow up in July 2011 were invited by mail to participate. The participation rate was 86%. Non-responders were contacted by telephone. A nurse interviewed all the participants about their diseases and self-reported current medication was recorded. Non-fasting blood samples were drawn, calcium, ionised calcium and intact parathyroid hormone were measured as previously described [2]. Subjects who had moved were interviewed by phone and blood samples were taken at their local PHCC. The ethics committee at Regional Ethical Review Board at Gothenburg University, (696–07) approved the study in 2008.

### Consent

Written informed consent was obtained from the patients for the publication of this report.

### Quality of life assessments

**Figure 2:**
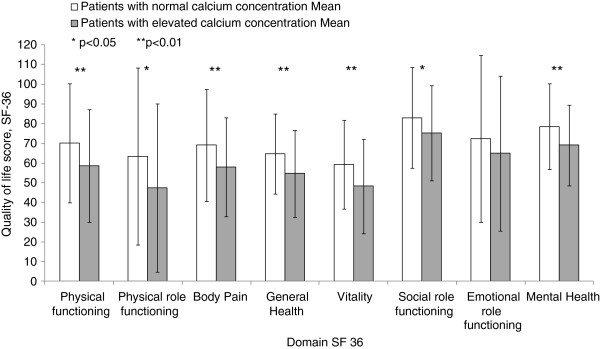
**Results of quality of life in the SF-36 survey.** Quality of life studied with SF-36 survey in patients with raised and normal calcium concentrations at Tibro Health Care Centre, Sweden. Higher scores indicate better quality of life.

The study participants completed the QoL health survey SF-36 [13], a health status measurement that allows comparison of the burden of illness. SF-36 is applicable regardless of patient condition and has been validated in a variety of diseases [14]. The survey has 36 simple questions, which are scored and aggregated. The survey defines eight separate and distinct areas or domains of health status: physical functioning, physical role functioning, bodily pain, general health, vitality, social role functioning, emotional role functioning and mental health, Figure 2. The number of questions that contributes to each domain ranges from 2 to 10. All scales are standardised from 0 to 100, with higher scores indicating better QoL.

Data from both comparison groups (patients with elevated calcium concentrations and patients with normal calcium concentrations) were compared with an age and gender-matched reference material (in average 2.3 individuals) from Sweden (n = 703) in the Swedish SF-36 national normative database [15]. We used 20-years intervals, (20–39, 40–59, 60–79, and 80–99 years) in the analyses.

### Statistics

Descriptive patient statistics were presented and comparisons were performed using the T-test, the Chi2-test and the Mann–Whitney U test depending of the type of data included in the analysis. The SF-36 survey results were analysed with non parametric test, substantially Mann–Whitney. A p-value < 0.05 was considered statistically significant. Data are given as mean ± SD in Figure 2 and as mean, 10th and 90th percentile in Tables 1 and 2. All statistical analyses were performed using the SPSS 20 statistical package.

**Table 1:** Characteristics of patients with elevated and normal calcium concentrations 1995–2000 and 2008–2010, men and women, at Tibro Health Care Centre, Sweden

	**Patients with elevated calcium concentrations**						**Patients with normal calcium concentrations**						**Comparison between the groups**		
	***Total***		***Men***		***Women***		***Total***		***Men***		***Women***		***Total p*-value**	***Men p*-value**	***Women p*-value**
**Variables**	**Mean**	**Percentile**	**Mean**	**Percentile**	**Mean**	**Percentile**	**Mean**	**Percentile**	**Mean**	**Percentile**	**Mean**	**Percentile**			
**Data from register 1995–2000**															
Number	127		28		99		254		56		198				
Age, January 1st 1995	61.4	36.6- 80.0	55.6	31.8- 77.0	63.0	38.8- 80.2	61.3	36.3- 80.0	55.5	39.0- 80.3	62.9	31.0- 77.0	n.s	n.s	n.s
Age, range	18–94		18–82		18–94		18–91		18–82		18–91				
S- Calcium^1^	2.66	2.56- 2.78	2.72	2.57- 2.88	2.64	2.56- 2.78	2.33	2.21- 2.43	2.31	2.19- 2.42	2.33	2.21- 2.44	<0.001	<0.001	<0.001
**Data from clinical investigation 2008–2010**															
Number	63		12		51		133		33		100				
Age, January 1st 2011	71.4	49.4- 89.0	67.7	49.2- 80.0	72.3	52.0- 89.0	70.0	45.2- 88.0	66.8	45.2- 84.4	71.0	45.8- 90.0	n.s	n.s	n.s
Age, range	31–97		45–82		31–97		30–99		31–99		30–97				
P- Calcium^2^	2.42	2.26- 2.59	2.39	2.18- 2.55	2.43	2.27- 2.64	2.35	2.22- 2.47	2.32	2.21- 2.42	2.36	2.23- 2.48	<0.001	0.085	0.003
Ionised Calcium^3^	1.29	1.22- 1.40	1.28	1.24- 1.31	1.29	1.22- 1.44	1.23	1.18- 1.27	1.22	1.16- 1.26	1.23	1.18- 1.28	<0.001	<0.001	<0.001
Parathyroid hormone^4^	68	20- 118	60	14- 136	69	21- 113	58	26- 107	57	22- 104	59	28- 113	n.s	n.s	n.s

Notes: Data are means (SD), n.s = not significant, Age in years. Reference range ^1^Serum Calcium analysed in whole blood (mmol/L) 2.15–2.55 (mmol/L), ^2^Plasma Calcium (mmol/L) 2.15–2.50, ^3^Ionised Calcium (mmol/L) 1.15–1.35, ^4^Parathyroid hormone (ng/L) 15–65.

**Table 2:** Hospitalisation of patients between 1st January 1967 and 31st December 2010 and contacts with Tibro Health Care Centre Sweden between 1st January 1998 and 31st December 2010 by patients with elevated and normal calcium concentrations 1995–2000, men and women

	**Patients with elevated calcium concentrations**						**Patients with normal calcium concentrations**						**Comparison between groups**		
	***Total***		***Men***		***Women***		***Total***		***Men***		***Women***		***Total p*-value**	***Men p*-value**	***Women p*-value**
**Variables**	**Mean**	**Percentile**	**Mean**	**Percentile**	**Mean**	**Percentile**	**Mean**	**Percentile**	**Mean**	**Percentile**	**Mean**	**Percentile**			
**Data from register**															
**Number**	127		28		99		254		56		198				
**Hospitalisation**															
Total occasion from 1967	11	2-21	14	2-33	10	2-20	8	2-18	7	1-14	9	2-19	0.017	0.002	n.s
Total days from 1967	75	6-179	82	4-214	73	7-178	58	4-146	39	3-98	63	6-161	n.s	0.006	n.s
Total occasion from 1995	7	2-13	11	2-29	7	1-12	5	1-11	5	1-11	6	1-11	0.029	0.015	n.s
Total days from 1995	51	3-128	61	4-154	48	2-128	37	3-77	26	3-63	41	3-79	n.s	0.018	n.s
**Diagnoses per individual from 1968**															
Cancer	1.3	0-4	1.7	0-6.3	1.2	0-3	0.9	0-2.2	0.8	0-3.6	1	0-2	0.003	n.s	0.017
Psychiatric diseases	0.9	0-2	0.7	0-2.3	0.9	0-2	0.5	0-1	0.3	0-1.6	0.5	0-1	n.s	n.s	n.s
Cardiovascular diseases	3.0	0-9	4.8	0-16	2.5	0-7	2.4	0-6	2.3	0-8	2.4	0-6	n.s	n.s	n.s
**Contact tibro HCC**															
**Physician consultations**															
Per individual	18.0	3.0- 35.4	12.5	2.6- 24.6	19.6	2.6- 39.0	17.7	4.0- 37.5	16.0	4.0- 31.0	18.2	3.5- 38.0	n.s	n.s	n.s
**Individuals visit psychosocial team**															
Number	13		4		9		14		1		13		n.s	0.02	n.s.

Notes: Percentile, 10th and 90th percentile n.s = not significant, HCC = Health Care Centre.

## Results

### Characteristics

Characteristics of patients with elevated and normal calcium concentrations are given in Table 1. The patients with elevated calcium concentrations at baseline had normalised calcium concentrations at the time of the follow up, however, still higher than in the group of patients with normal calcium concentrations at base-line, Table 1. The two groups demonstrated similar values in the upper reference range of parathyroid hormone.

There was no difference in marital status, level of education, or employment, number of smokers between patients with elevated and normal calcium concentrations (data not shown).

### Quality of life

The group with elevated calcium concentration had lower scores in all SF-36 domains compared with the group with normal calcium concentration, Figure 2. In five domains the scores were at least 10 points lower in the group with elevated calcium concentrations compared with the patients with normal calcium concentrations, Figure 2. The differences were significant in all domains, except emotional functioning, Figure 2. Compared with the Swedish norm group patients with elevated calcium concentrations had significantly lower scores in all SF-36 survey domains except bodily pain.

### Health care consumption

At the time of follow up in July 2011 only 21 of the patients with elevated calcium concentrations were of working age, the rest had retired or died. For the overall group there were significantly more days in inpatient care compared with the normal calcium group, Table 2. Looking at the subpopulation of men, the difference was not significant, although numerically the group with elevated calcium concentrations had twice as many hospital days and care occasions as the group with normal calcium concentrations. Patients with elevated calcium concentrations had significantly more cancer diagnoses associated with hospitalisation than patients with normal calcium concentrations. We could not find any differences between the groups in number of visits to the PHCC physician; however, men with elevated calcium concentrations had more contacts with the psychosocial team, Table 2.

Overall patients with elevated calcium concentrations had significantly more sick leave and drug treatment, compared with patients with normal calcium concentrations, Table 3.

**Table 3:** Sick leave between 4th November 2003 and 27th December 2010 and medication of patients with elevated and normal calcium concentrations 1995–2000 at Tibro Health Care Centre, Sweden

	**Patients with elevated calcium concentrations**			**Patients with normal calcium concentrations**			**Comparison between the groups**
**Variables**	**Number**	**Mean**	**Median**	**Number**	**Mean**	**Median**	**p-value**
**Data from register**							
Number	127			254			
Sick leave							
Individuals with certificates	12			25			0.90
Certificates/individual (n)		7.5	6.5		5.2	2.0	0.02
Days/individual		258	144		120	22	0.007
Days/certificate		34	28		23	17	< 0.001
Days/sick leave episode		87	47		54	15	0.001
**Data from clinical investigation**							
Medication							
All	63	5.3	5.0	132	3.5	3.0	0.002

n.s = not significant.

## Discussion

To our knowledge this is the first study showing that patients in primary care with elevated calcium concentrations per se have significantly reduced QoL compared with patients with normal calcium concentrations and more pronounced reduction compared with the Swedish norm population. They had significantly more contacts with the psychosocial team, more sick leaves, more medication and more hospitalisations than those with normal calcium concentrations.

### Quality of life

In many of the domains in the SF-36 health survey the group with elevated calcium concentrations had more than 10 points lower results compared with the Swedish norm. This magnitude of difference has probably a clinical significance at group level [2].

It may seem counter-intuitive that the QoL according to SF-36 survey showed a significant difference between the two groups despite the fact that the mean calcium concentration in the group with elevated calcium concentration at baseline had normalised at the time of follow up. However, the highest calcium values at baseline were found among those who died during the observation period, and almost half of the 46 patients with pHPT had undergone parathyroidectomy (and in most cases became normocalcaemic after surgery) [2]. This contributes to the decline of calcium concentrations at group level. It is also reasonable to assume that patients who did not participate in the follow up may be in the poorer spectrum of health, one third of the drop-outs reported disease as a reason not to participate in the follow up. Our interpretation is that the differences found in the SF-36 scores may in fact underestimate the QoL in hypercalcaemic patients.

There were no significant difference in PTH values between patients with elevated and normal calcium concentration. We believe that the 20 patients that had undergone parathyroidectomy contribute to the decline in PTH levels in the group with elevated calcium concentrations.

### Health care consumption

It is previously known that low scores in the domain general health is associated with more doctor calls, more medication and hospitalisation [16]. This is supported by our study as we found an increased health care consumption in patients with elevated calcium concentrations.

The subjects eligible for sick leave were few because of the age distribution in the study, but still, significantly more certificates and more sick leave days were found in the group with elevated calcium concentration. This is in line with a Swedish study of women with unknown, mild pHPT where an increased sick leave rate was observed in the years before the diagnosis [9,10]. Had it been possible to compare our material with a Swedish norm regarding sick leave, we speculate that we may have seen an even greater difference since our controls were patients with symptoms that led to ordering of calcium analyses and not healthy controls. Furthermore, there was a low score in the SF-36 domain physical functioning, a domain which has previously been shown to be correlated to a reduced capacity to work [15].

Significantly more individuals were hospitalised with cancer diagnoses among those with elevated calcium concentrations. The difference is not significant in the male subpopulation, probably because of the small numbers. In our previous study we found increased mortality among men, especially in cancer [2]. We could not find any differences between the groups in number of visits to the PHCC physicians in line with the Swedish screening study [9,10]. However men with elevated calcium concentrations had more contact with the psychosocial team.

The strength of this study is the high participation rate, 86% at follow up, probably due to the confinement of the study to one PHCC. Access to inpatient registry from all of Sweden for several decades means that the material is comprehensive. This study is unique since there is no previously published study of hypercalcaemia per se and QoL.

One limitation of this study was that the inclusion of the calcium analysis for the patient group was based on a single calcium concentration measurement which was corrected for calcium in 75% of cases. This may have led to that erroneous individuals were included in the study. Another limitation of this study is that SF-36 data at baseline is not available for comparison with SF-36 data at follow up. Another drawback is also that the controls were selected among patients with various disorders at the PHCC. An age and sex matched random sample from the population would have been optimal, but it was not possible since the study which is the basis for this prospective study was retrospective. Another limitation is the small study sample as the study was performed in a single PHCC. The results should therefore be interpreted with caution especially, in the subgroups.

## Conclusions

In conclusion, elevated calcium concentrations are associated with significantly reduced QoL and increased health care consumption, as measured by, sick leave, drug prescriptions and the number of visits and admissions to health care centres and hospitals. The clinical consequences of poor QoL in patients with elevated calcium concentrations include not only suffering for the individual, but also an increased cost to society in terms of increased health care consumption. Elevated calcium concentration is therefore an important warning flag that should alert the physician to further investigate and care for the patient.

## Abbreviations

QoL: Quality of life; PHCC: Primary health care centre; pHPT: Primary hyperparathyroidism; SF-36: Medical outcome study 36 item short form health survey.

## Competing interests

The authors declare that they have no competing interests.

## Authors’ contributions

SD conceived the study, drafted the manuscript, responded to the reviewer comments and critically revised the manuscript. RE conceived the study and critically revised the manuscript for important intellectual content. PH performed some of the statistical analyses and critically revised the manuscript. SJ participated in the design of the study and critically revised the manuscript for important intellectual content. KBB conceived the study, responded to the reviewer comments and critically revised the manuscript. All authors read and approved the final manuscript.

## Pre-publication history

The pre-publication history for this paper can be accessed here:

http://www.biomedcentral.com/1471-2296/15/84/prepub
